# A severe neurodegenerative disease with Lewy bodies and a mutation in the glucocerebrosidase gene

**DOI:** 10.1038/s41531-023-00501-4

**Published:** 2023-04-05

**Authors:** Jussi O. T. Sipilä, Laura Kytövuori, Tuomas Rauramaa, Hugo Rauhamaa, Valtteri Kaasinen, Kari Majamaa

**Affiliations:** 1 Clinical Neurosciences, University of Turku, Turku, Finland; 2 Department of Neurology, Siun Sote North Karelia Central Hospital, Joensuu, Finland; 3 Research Unit of Clinical Medicine and Medical Research Center Oulu, Oulu University Hospital and University of Oulu, Oulu, Finland; 4 Neurocenter, Neurology, Oulu University Hospital, Oulu, Finland; 5 Unit of Pathology, Institute of Clinical Medicine, University of Eastern Finland, Kuopio, Finland; 6 Neurocenter, Turku University Hospital, Turku, Finland

**Keywords:** Parkinson's disease, Clinical genetics, Clinical genetics, Neurodegeneration

## Abstract

Several heterozygous variants of the glucocerebrosidase gene (*GBA1*) have been reported to increase the risk of Parkinson’s disease (PD) and dementia with Lewy bodies (DLB). *GBA1*-associated PD has been reported to be more severe than idiopathic PD, and more deleterious variants are associated with more severe clinical phenotypes. We report a family with a heterozygous p.Pro454Leu variant in *GBA1*. The variant was associated with a severe and rapidly progressive neurodegenerative disease with Lewy bodies that were clinically and pathologically diverse. Pathogenicity prediction algorithms and evolutionary analyses suggested that p.Pro454Leu is deleterious.

## Introduction

Several heterozygous variants of the glucocerebrosidase gene (*GBA1*) have been associated with an increased risk of Parkinson’s disease (PD) and dementia with Lewy bodies (DLB)^1,2^. Recent studies have shown that the frequency of any *GBA1* variant is 8–15% in European or North American PD patients^3–7^, but the higher-frequency, lower-penetrance alleles p.Glu365Lys (legacy name E326K) and p.Thr408Met (T369M) contribute most to the reported frequencies. The higher-penetrance alleles include p.Asn409Ser (N370S) and p.Leu483Pro (L444P) and they make 3.1–6.4% of the proportion^8^. Patients with pathogenic *GBA1* variants have been reported to present with an earlier onset and a more severe clinical course compared to patients with idiopathic PD (iPD), but the results are not uniform^9–13^. On the other hand, reports on neuropathological findings associated with clinical data in GBA1-PD are scarce^14,15^. A compound heterozygous patient with p.[Pro454Arg];[Leu483Pro] and with Gaucher disease (GD) has previously been reported^16^, and the heterozygous p.Pro454Arg (P415R) variant has been described in PD^17^. Here we report clinical and neuropathological findings in two siblings harboring a p.Pro454Leu variant in *GBA* and presenting with a severe neurodegenerative disease.

## Results

### Clinical case presentations

**Fig. 1:**
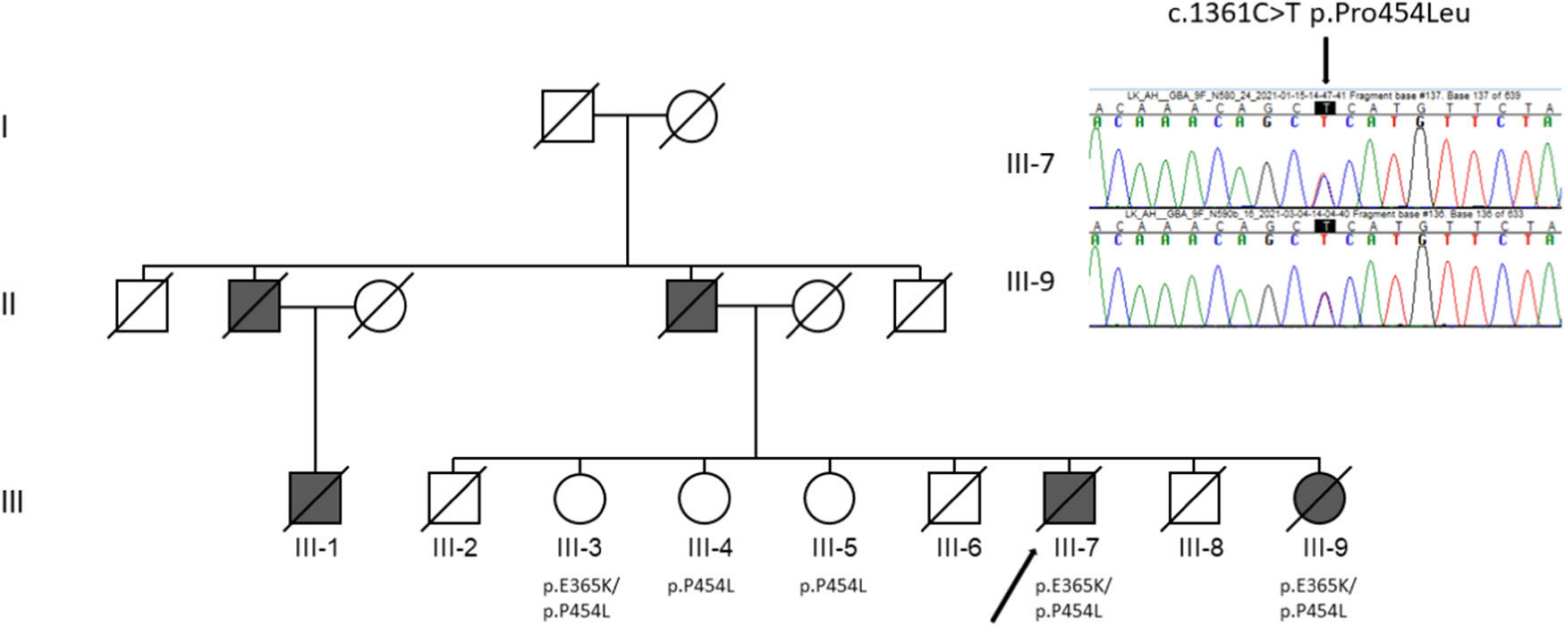
The pedigree of the affected family. Arrow, the proband; open symbols, healthy persons; solid symbols, affected persons. Chromatograms show the heterozygous nucleotide change leading to the variant p.Pro454Leu.

The proband (III-7 in Fig. 1) had previously been diagnosed with hypercholesterolemia, type 2 diabetes, atrial fibrillation, and bradycardia necessitating a cardiac pacemaker. At the age of 68 years, he presented with muscle stiffness, hypomimia, dysphagia, hypophonia, trouble initiating speech, and disturbed sleep. On examination, increased muscle tone without tremor was detected, and his balance was normal. Mini-Mental State Examination (MMSE) score was 26/30. Non-contrast head CT was normal, but dopamine transporter (DAT) SPECT imaging revealed a symmetric loss of tracer binding in the posterior putamen and a slight decrease of binding in the caudate nucleus. Polysomnography indicated a sleep apnoea with a central component and electrodiagnostic examination revealed a demyelinating motor polyneuropathy. He was diagnosed with PD and levodopa and pramipexole were initiated with modest effect. He developed a slight resting tremor during follow-up.

At age 70 years he was admitted to hospital because of disorientation and hallucinations and a reported seizure. Severe orthostatic hypotension, worsened by levodopa, was regarded as the cause of seizure-like symptoms. Three months later he was admitted because of pneumonia and, by this time, he had developed constant drooling and myoclonic jerks. After discharge, he was cognitively intact according to his wife, was continent and could walk some 600 meters at his best. However, he became more dysarthric, his gaze became staring and ocular movements were found to be restricted. On occasions the only remaining active eye movement was minimal upgaze and at times he was able to perform vertical pursuit, although this movement was frequently broken. His cognition declined and hypotension worsened. Bradykinesia and hypokinesia progressed with ambiguous response to levodopa, which was then tapered. He died of pneumonia three and a half years after presentation.

The younger sister (III-9) of the proband was diagnosed with unspecified dementia and parkinsonism at the age of 65 years. She had been healthy apart from hypercholesterolemia but had developed progressive cognitive problems during the previous 2–3 years. At the first visit, MMSE score was 15/30 and she had mild depression. On clinical examination mild bradykinesia, hypomimia and slight rigidity were observed. Orthostatic test was carried out repeatedly, but it was positive only once. There was mild temporal atrophy and slight hippocampal atrophy in non-contrast head CT. DAT SPECT revealed a bilateral loss of posterior putamen signals and a decrease in caudate signals. Cerebrospinal fluid levels of tau, phosphorylated tau and amyloid beta(1–42) were normal.

At the age of 67 years, she was completely dependent on her spouse. She walked with short steps and stooped forward, and there was clear rigidity in upper extremities but no tremor. She had sialorrhea and severe apraxia. Eye movements were slow but with full range in all directions. levodopa and memantine proved ineffective, while donepezil increased alertness but only briefly. At the age of 68 years her MMSE score was 10/30 and she had frequent falls. The final visit was 7 months later, when she was still able to speak, but had severe dysarthria together with sialorrhea, and rigidity. Her spouse reported on symptoms consistent with rapid eye movement (REM) sleep behavior disorder. She died of pneumonia three and a half years after the diagnosis.

### Neuropathology

**Fig. 2:**
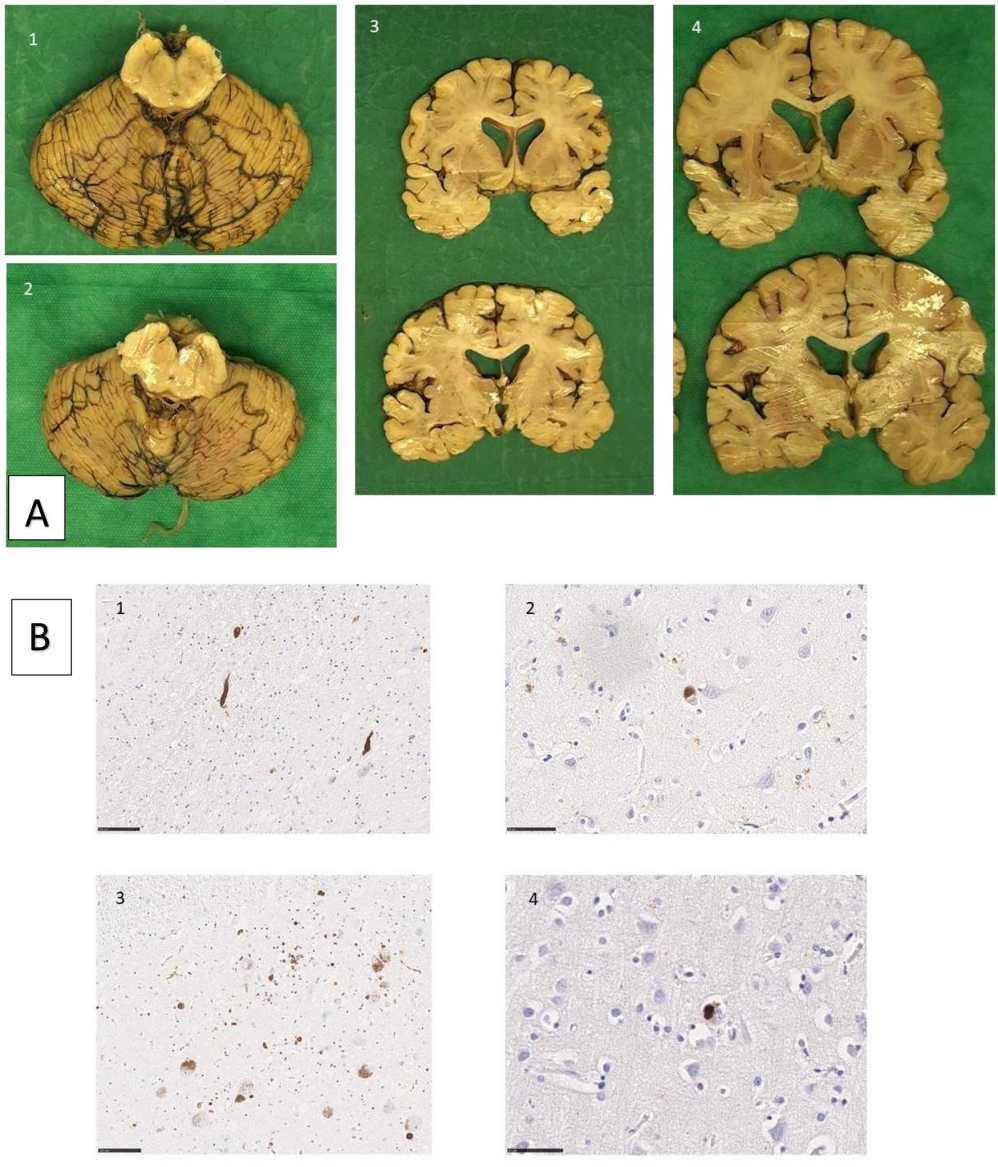
Neuropathological images. **A** Macroscopic images. 1. Substantia nigra and cerebellum (proband). 2. Substantia nigra and cerebellum (sister). 3. Coronal sections including basal ganglia (proband). 4. Coronal section including basal ganglia (sister). **B** Microscopic images. 1. Lewy neurites (substantia nigra, proband). 2. Cortical lew body (proband). 3. Lewy bodies and neurites (substantia nigra, sister). 4. Cortical Lewy body, sister.

The brain of the proband weighed 1492 g and his sister’s 1403 g. Both patients had extensive Lewy body pathology that, however, was not found in the basal ganglia of the proband (Table 1 and Fig. 2). No pathological changes were observed in the cerebellum, white matter, or dentate nucleus. The proband had hyperphosphorylated τ pathology consistent with primary aging-related tauopathy (PART) as well as cerebral amyloid angiopathy (CAA).

**Table 1:** Clinical and neuropathological features of the patients.

	Proband	Sister
Sex	Male	Female
Age of onset (years)	66	63
Age of death (years)	71	68
Levodopa response	Yes	No
Parkinsonism phenotype	Akinetic-rigid	Akinetic-rigid
Motor asymmetry	No	No
RBD	No	Yes
Dyskinesias	No	No
Dystonia	No	No
Ophthalmoplegia	Yes	No
Hallucinations	Only with scopolamin patch	No
Tendon reflexes	Relatively brisk	Relatively brisk
Plantar response	Bilaterally positive	Bilaterally negative
Spasticity	No	No
Orthostatic testing	Positive	Negative
Bladder dysfunction	No	No
Bowel dysfunction	Slight	Yes
Erectile dysfunction	No	n.a.
Sialorrhea	Yes	Yes
Polyneuropathy	Motor demyelinating	No
Cerebrospinal fluid	n.a.	Eryt: 8 (normal 0) Leuk: 1 (normal 0–5) Prot: 213 (normal 255-620) BAm42: 626 (normal > 500) Tau: 149 (normal < 400) FosTau: 26 (normal < 70)
*Macroscopy*		
Substantia nigra	Neuronal loss	Neuronal loss
Hippocampus	-	Mild atrophy
Cerebral arteries	Mild atherosclerosis	Mild atherosclerosis
*Microscopy*		
Hyperphosphorylated τ	PART, CA2++	Scattered tangles, CA2++
Amyloid β	Small number of diffuse cortical plaques	–
*α-synuclein pathology*		
Subtantia nigra	+++	+++
Pons	+++	+++
Locus coeruleus	+++	+++
Dorsal motor nucleus of vagus	+++	+++
Basal ganglia	–	+++
Cortex	++	++

*Bam42* Beta Amyloid 42 (pg/ml), *CA2* Cornu Ammonis 2 area of the hippocampus, *Eryt* erythrocytes (×1,000,000); *FosTau* Phosphorylated tau protein (pg/ml), *Leuk* leukocytes (×1,000,000), *n.a.* not available, *PART* primary aging-related tauopathy, *Prot* proteins (mg/l), *RBD* REM sleep behavior disorder. – none, ++ moderate, +++ severe, *Tau* total tau protein (pg/ml).

### Genetic findings

A heterozygous p.Pro454Leu variant in *GBA1* (NM_000157.4:c.1361C>T, GRCh38: 1-155235708-G-A, Fig. 3) was found in the two affected siblings and in their three healthy elder sisters. In addition, the affected siblings and the healthy sister III-3 harbored p.Glu365Lys and it was confirmed that they were compound heterozygotes p.[Glu365Lys];[Pro454Leu]. The siblings reported that their father and paternal uncle had had PD (Fig. 1) and that the son of the affected uncle had had similar symptoms, but they were not aware that any diagnosis had been set. DNA was not available from the affected relatives. The family had been native Finns for several generations.

In silico analysis by using a consensus classifier PredictSNP suggested that the p.Pro454Leu variant is deleterious^18^. Proline-454 is the N-terminal residue in the eighth α-helix, 454-PMFYHLGHFS-463, of the triosephosphate isomerase barrel in the catalytic domain of GBA1^19^. The helix and the proline residue at position 454 are highly conserved among 110 species (https://alignmentviewer.org).

## Discussion

We found a p.Pro454Leu *GBA1* variant in two siblings with a neurodegenerative phenotype, and the family history suggested that two more individuals had been affected in the previous generation. The disease was severe in the two siblings despite some differences in their clinical features and neuropathological findings. The penetrance of the phenotype was incomplete, as the three elder siblings were unaffected. Among the variants reported in *GBA* p.Pro454Leu appears to be particularly deleterious.

The age of onset was similar to that reported in GBA1-PD patients^4,5^, whereas disease duration was shorter being only 2 years^5,14,20–22^. In addition, the clinical phenotype differed markedly between the siblings (Table 1). The phenotype of the proband initially corresponded with iPD, but atypical features accumulated, levodopa response became more and more ambiguous, and his cognition became affected as the disease progressed. Peripheral neuropathy and the signs suggesting upper motor neuron damage remained unexplained. The phenotype of the sister included a major cognitive involvement and mild extrapyramidal findings suggesting the diagnosis of DLB. However, several characteristic features, such as hallucinations, REM sleep behavior disorder, fluctuating cognition, and variations in attention and alertness were lacking and her response to donepezil was minimal. Neuropathological differences included lack of Lewy body pathology in the basal ganglia of the proband, which might explain the differences in the cognitive phenotype^23^. Overall, the neuropathological findings did not differ from those in iPD, which is consistent with previous data^24,25^. Poor levodopa response has been reported in both iPD and GBA1-PD, and neuropathology correlates inadequately with levodopa response^21,26–28^. The most fitting description of this GBA1 p.Pro454Leu disorder would be a rapidly progressive disease with Lewy bodies^22,29^.

The severity of *GBA1* variants influences the PD phenotype. Motor, cognitive, olfactory and psychiatric symptoms have been reported to be more severe in PD patients with the severe *GBA1* variants causing neuronopathic GD (type II GD) compared to those with the mild *GBA1* variants causing non-neuronopathic GD^30^. Interestingly, a patient with type II GD has previously been reported with the p.Pro454Arg variant in trans with the severe p.Leu483Pro variant making the patient a compound heterozygote p.[Pro454Arg];[Leu483Pro]^16^. The p.Pro454Arg variant has been shown to significantly alter both RNA and protein levels in mutant cell lines leading to a severe decrease in β-glucocerebrosidase activity^16,31,32^. The severity of the variants cannot be evaluated solely based on decreased glucocerebrosidase activity, as a recent study has shown that the activity does not seem to be associated with PD risk and severity^33^. Therefore, the mechanisms from mutation to pathology and phenotype remain unclear and may include novel pathways, such as general regulatory functions of *GBA1*^34^. Interestingly, the p.Pro454Arg variant has been shown to alter the transport of the β-glucocerebrosidase enzyme to lysosomes in contrast to common GD variants^35^. Furthermore, the pathogenicity prediction algorithms and evolutionary analyses suggested that both p.Pro454Arg and p.Pro454Leu are deleterious.

The affected siblings and the eldest healthy sister were compound heterozygotes harboring p.[Glu365Lys];[Pro454Leu]. The p.Glu365Lys variant is common in the Finnish population with an allele frequency of 4.3 % (gnomAD v2.1.1). A slightly increased frequency of the variant has been reported among patients with PD^36^, but subjects with homozygous p.Glu365Lys do not present with GD. A mild functional defect has been found in some studies^37^, but p.Glu365Lys appears to be only a weak risk factor of PD^38^. Patients with p.Glu365Lys do not differ considerably clinically from patients with iPD, but they appear to have more cognitive problems than non-carriers^39^. Therefore, it is extremely unlikely that p.Glu365Lys is a potent contributor to the phenotype, but a minimal additional effect cannot be ruled out.

In conclusion, we found a p.Pro454Leu *GBA1* variant in two siblings with a neurodegenerative phenotype. Pathogenicity prediction algorithms and evolutionary analyses suggested that p.Pro454Leu is deleterious. The siblings presented with a severe and diverse neurodegenerative phenotype and with Lewy body pathology. The penetrance of the phenotype was incomplete.

## Methods

The brains were stored in 10 % buffered formaldehyde for at least 1 week before being cut into 1-cm-thick coronal slices and assessed macroscopically by a neuropathologist. The samples were taken from 16 regions (frontal cortex, temporal cortex, cingular gyrus at the level of mamillary bodies, parietal cortex, motor cortex, occipital cortex, anterior and posterior hippocampus with the entorhinal cortex, basal forebrain including amygdala, striatum, thalamus, midbrain, pons, medulla, cerebellar vermis and cortex) and embedded in paraffin. Immunohistochemically analyses were made for 42/α-synuclein, BD Transduction Laboratories 1:3000, AT8, Innogenetics 1:750, and 6F/3D, Dako/Agilent, 1:100.

Blood DNA was extracted using standard methods. Compound heterozygosity of p.Glu365Lys and p.Pro454Leu was confirmed by amplification of DNA fragment containing both variants and allele-specific restriction analysis followed by gel extraction and Sanger sequencing of purified fragments. Exome sequencing was carried out as described previously^40^, and a panel of 22 PD-related genes was analyzed for single nucleotide variants and copy number variants^41^.

This study was approved by the Regional Ethics Committee, Northern Ostrobothnia Hospital District (PD-NEF etmk 51/2017). All siblings gave their written informed consent to participate in this study.

### Reporting Summary

Further information on research design is available in the Nature Research Reporting Summary linked to this article.

## Supplementary information

Reporting-summary

## Data Availability

Genotype and clinical phenotype data are available in the manuscript. Variant has been submitted to ClinVar (SCV002587825).
